# Granulosa cell proliferation is inhibited by PGE2 in the primate ovulatory follicle

**DOI:** 10.1080/19768354.2020.1764385

**Published:** 2020-05-21

**Authors:** Patric S. Lundberg, Gil J. Moskowitz, Carmel Bellacose, Esra Demirel, Heidi A. Trau, Diane M. Duffy

**Affiliations:** a Department of Microbiology and Medical Molecular Biology, Eastern Virginia Medical School, Norfolk, VA, USA; b Department of Department of Computer Science, Old Dominion University, Norfolk, VA, USA; c Department of Physiological Sciences, Eastern Virginia Medical School, Norfolk, VA, USA

**Keywords:** PGE2, ovulation, granulosa cell, cell cycle, Ingenuity Pathway Analysis

## Abstract

Prostaglandin E2 (PGE2) is a key paracrine mediator of ovulation. Few specific PGE2-regulated gene products have been identified, so we hypothesized that PGE2 may regulate the expression and/or activity of a network of proteins to promote ovulation. To test this concept, Ingenuity Pathway Analysis (IPA) was used to predict PGE2-regulated functionalities in the primate ovulatory follicle. Cynomolgus macaques underwent ovarian stimulation. Follicular granulosa cells were obtained before (0 h) or 36 h after an ovulatory dose of human chorionic gonadotropin (hCG), with ovulation anticipated 37–40 h after hCG. Granulosa cells were obtained from additional monkeys 36 h after treatment with hCG and the PTGS2 inhibitor celecoxib, which significantly reduced hCG-stimulated follicular prostaglandin synthesis. Granulosa cell RNA expression was determined by microarray and analyzed using IPA. No granulosa cell mRNAs were identified as being significantly up-regulated or down-regulated by hCG + celecoxib compared with hCG only. However, IPA predicted that prostaglandin depletion significantly regulated several functional pathways. Cell cycle/cell proliferation was selected for further study because decreased granulosa cell proliferation is known to be necessary for ovulation and formation of a fully-functional corpus luteum. Prospective in vivo and in vitro experiments confirmed the prediction that hCG-stimulated cessation of granulosa cell proliferation is mediated via PGE2. Our studies indicate that PGE2 provides critical regulation of granulosa cell proliferation through mechanisms that do not involve significant regulation of mRNA levels of key cell cycle regulators. Pathway analysis correctly predicted that PGE2 serves as a paracrine mediator of this important transition in ovarian structure and function.

## Introduction

The midcycle surge of luteinizing hormone (LH) initiates structural and functional changes within the ovulatory follicle which culminate in release of the oocyte and formation of the corpus luteum (Duffy et al. 2019). Follicular prostaglandins are low prior to the LH surge. The LH surge initiates follicular synthesis of prostaglandins and, in particular, prostaglandin E2 (PGE2) (Sirois 1994; Sirois and Dore 1997; Mikuni et al. 1998; Duffy and Stouffer 2001). Locally-elevated PGE2 is a critical mediator of ovulatory changes including expansion of cumulus granulosa cells, angiogenesis, follicle rupture, and release of an oocyte capable of fertilization (Duffy 2015; Trau et al. 2015). Blockade of PGE2 synthesis or action results in unruptured follicles with retained oocytes (Hizaki et al. 1999; Tilley et al. 1999; Duffy and Stouffer 2002).

PGE2 plays a critical role in ovulation. Perhaps surprisingly, few PGE2-regulated gene products have been identified in the ovulatory follicle. Gene array analyses focusing on gene expression regulated by the LH surge (or hCG in experimental models) have identified numerous gonadotropin-regulated genes in ovarian cells and tissues from rodents, domestic animals, monkeys, and humans (Fan et al. 2009; Xu et al. 2011; Christenson et al. 2013; Wissing et al. 2014). Since the LH surge initiates synthesis of ovulatory prostaglandins, we might expect that prostaglandin-regulated genes should be a subset of all LH-regulated genes. A few prostaglandin-regulated genes have been identified in prospective studies of LH-regulated genes (Seachord et al. 2005; Li Q et al. 2006; Sayasith et al. 2008; Markosyan and Duffy 2009). Additional prostaglandin-regulated gene products have been identified after ovulatory dysfunctions were noted in mice lacking expression of the key prostaglandin synthesis enzyme PTGS2 (Hizaki et al. 1999; Tilley et al. 1999). However, the well-established failure of ovulation in the absence of prostaglandins cannot reasonably be explained by regulation of a such a small number of gene products.

The goal of this study was to use pathway analysis to identify prostaglandin-regulated functionalities of granulosa cells. While changes in functionalities may be secondary to altered gene expression, functionalities may also be linked to prostaglandin-dependent changes in the function of existing proteins. Ex vivo gene expression analysis was performed using granulosa cells obtained from monkeys before and after an ovulatory gonadotropin stimulus; additional granulosa cells were obtained after an ovulatory gonadotropin stimulus in combination with the PTGS2 inhibitor celecoxib. Unbiased analysis identified specific pathways that showed significant predicted functional changes with PGE2 depletion. Follow-up studies demonstrated that PGE2 reduced cell cycle progression by granulosa cells, an essential step in the transition from preovulatory follicle to corpus luteum. Our studies indicate that PGE2 provides critical regulation of granulosa cell proliferation through mechanisms that do not require a significant change in the mRNA level of any individual cell cycle regulator. The literature does provide precedent for the concept that subtle changes in many gene products results in measurable, functional changes in cell function. Pathway analysis correctly predicted that PGE2 serves as a paracrine mediator of this important transition in ovarian structure and function. Overall, these studies demonstrate the utility of pathway analysis to identify ovulatory pathways regulated by PGE2 and other paracrine mediators of ovulation.

## Materials and methods

### Monkey ovarian cells and tissues

Granulosa cells and whole ovaries were obtained from adult female cynomolgus macaques (*Macaca fascicularis*) aged 4–10 years, 3–4.5 kg, at Eastern Virginia Medical School (EVMS). All animal protocols and experiments were approved by the EVMS Animal Care and Use Committee and were conducted in accordance with the National Institutes of Health Guide for the Care and Use of Laboratory Animals. Animal husbandry and sample collection were performed as previously described (Seachord et al. 2005). Briefly, blood samples were obtained under ketamine chemical restraint by femoral venipuncture, and serum was stored at –20°C. Aseptic surgeries were performed in a dedicated surgical suite under isoflurane anesthesia, and post-operative pain was controlled with buprenorphine alone or in combination with ketoprofen.

A controlled ovarian stimulation model developed for the collection of multiple oocytes for *in vitro* fertilization was used to obtain monkey granulosa cells (Seachord et al. 2005). Beginning within 3 days of initiation of menstruation, FSH (90 IU daily, Merck and Co., Inc., Kenilworth, NJ) was administered for 6–8 days, followed by daily administration of 90 IU FSH plus 60 IU LH (Serono Reproductive Biology Institute, Rockland, MA) for 2 days to stimulate the growth of multiple preovulatory follicles. A GnRH antagonist (Antide,0.5 mg/kg body weight; Serono) was also administered daily to prevent an endogenous ovulatory LH surge. Adequate follicular development was monitored by serum estradiol levels and ultrasonography. Follicular aspiration was performed during aseptic surgery before (0 h) or up to 36 h after administration of 1000 IU r-hCG (EMD Serono). To inhibit follicular prostaglandin production during the ovulatory interval, additional animals were treated with gonadotropins and Antide as described above; these animals also received the PTGS2 inhibitor celecoxib (Celebrex, Pfizer, NY; 6.7 mg/kg body weight orally every 12 h) beginning with hCG administration until follicles were aspirated 36 h later (Seachord et al. 2005). At aspiration, each follicle ≥4 mm in diameter was pierced with a 22-gauge needle, and the contents of all aspirated follicles were pooled. Ovulatory follicles in cynomolgus macaques are typically 4–6 mm in diameter as assessed by ultrasonography and confirmed by direct measurement at surgery; ovulation is anticipated 37–40 h after hCG in this species. Whole ovaries were also obtained from monkeys experiencing ovarian stimulation as described above. Ovaries were bisected, maintaining at least two follicles greater than 4 mm in diameter on each piece. Ovarian pieces were fixed in 10% formalin for paraffin sections.

### Monkey granulosa cell RNA, Affymetrix array, and Ingenuity Pathway Analysis

Monkey granulosa cells and oocytes were pelleted from the follicular aspirates by centrifugation at 250 X g. Following oocyte removal, a granulosa cell-enriched population of cells was obtained by Percoll gradient centrifugation (Seachord et al. 2005); viability was assessed by trypan blue exclusion and averaged 85%. Granulosa cells were stored at –80°C until RNA was isolated using Trizol reagent. Total RNA from monkey granulosa cell obtained at 0 h hCG, 36 h hCG, and 36 h hCG + celecoxib were processed using the Affymetrix GeneChip WT PLUS Reagent Kit and hybridized to Gene Atlas Cynomolgus Gene 1.1 ST Arrays Strips, and Robust Multichip Average (RMA)-normalized (Irizarry et al. 2003). Gene IDs from Macaca fascicularis (GCA_000364345.1 2013/06/12 version) was used to verify transcripts reported in this study. Gene Level expression data (*p*<0.05 threshold) was imported to Ingenuity Pathway Analysis (IPA, Germantown, MD) software for analysis. Expression ratios were generated, ratios of gene expression data were overlaid on expressed genes in networks, and the IPA Molecule Activity Predictor was used to color-code upstream regulators.

### Human granulosa cells

Granulosa cells were obtained from healthy women undergoing ovarian stimulation for oocyte donation at the Jones Institute for Reproductive Medicine at EVMS. The Institutional Review Board at EVMS determined that this use of discarded human granulosa cells does not constitute human subjects research as defined by 45 CFR 46.102(f). Follicular aspirates are routinely collected 34–36 h after administration of an ovulatory dose of hCG. Granulosa cells were transferred to our laboratory after oocyte removal. A granulosa cell-enriched population of cells was obtained by Percoll gradient centrifugation as described above for monkey granulosa cells. Cells were cultured on fibronectin-coated chamber slides (Nalgene Nunc, Rochester, NY) in DMEM/F12 media (Sigma) containing 10% fetal bovine serum (Atlanta Biologicals), penicillin (100 U/ml), streptomycin (100 µg/ml), insulin (10 µg/ml), transferrin (10 µg/ml), and selenium (10 ng/ml). Media were changed each day for 7 days as has been shown to allow these granulosa cells to regain gonadotropin responsiveness comparable to granulosa cells obtained from large, preovulatory follicles (Freimann et al. 2004; Al-Alem et al. 2015). To initiate treatments, fresh media was supplemented with final concentrations of hCG (20 IU/ml, Sigma), the general cyclooxygenase inhibitor indomethacin (0.1 μM, Sigma), and/or PGE2 (0.1 μM, Cayman). After 24 or 48 h in vitro, media was removed, cells were fixed in 10% formalin for 20 min, and slides were stored in phosphate buffered saline at 4C until used for immunodetection of Ki67.

### Quantitative RT-PCR (qPCR)

Determination of granulosa cell levels of individual mRNAs was conducted by qPCR using a Roche LightCycler (Roche Diagnostics, Indianapolis, IN). Total RNA was obtained from granulosa cells using Trizol reagent, treated with DNase and reverse transcribed as previously described (Duffy, Dozier, et al. 2005). PCR was performed using the FastStart DNA Master SYBR Green I kit (Roche) following manufacturer’s instructions. Primers were designed using Primer-Blast (NCBI; http://www.ncbi.nlm.nih.gov/tools/primer-blast) based on available human or monkey sequences and span an intron to prevent undetected amplification of genomic DNA (Table 1). PCR products were sequenced (Genewiz, South Plainfield, NJ) to confirm amplicon identity. At least 5 log dilutions of the sequenced PCR product were included in each assay and used to generate a standard curve. For each sample, the content of mRNA of interest and *ACTB* mRNA was determined in independent assays. No amplification was observed when cDNA was omitted. All data were expressed as the ratio of mRNA of interest to *ACTB* mRNA for each sample.

**Table 1.** Primers for qPCR.

Target		Primer sequence (5′–3′)	Accession number
ACTB	Up	ATCCGCAAAGACCTGT	NM_001285025.1
	Down	GTCCGCTAGAAGCAT	
CCNB1	Up	ACCTGATGGAACTAACTATGT	NM_031966.2
	Down	GTGCTTTGTAAGTCCTTGAT	
CCNE1	Up	CAGCCCCATCATGCCGA	NM_001238
	Down	TCACACACCTCCATTAACCAA	

#### Immunodetection of Ki67

Deparaffinized monkey ovarian sections and human granulosa cells on chamber slides were used for immunodetection of the proliferation antigen Ki67 essentially as previously described (Duffy, Seachord, et al. 2005). Briefly, a mouse monoclonal antibody against human Ki67 (Dako, Glostrup, Denmark) was used at a 1:100 dilution (final concentration 46 µg/L); immunodetection was achieved using a biotinylated anti-mouse IgG ABC kit (Vector Laboratories, Burlingame, CA) and visualized with DAB (brown stain, Vector). In some experiments, the primary antibody was omitted as a negative control. All slides were counterstained with hematoxylin. To quantify Ki67, each tissue section or chamber was imaged 4 times in a predetermined standard pattern using an Olympus BX41 microscope fitted with a DP70 digital camera and associated software (Melville, NY). For each image, nuclei with brown stain (Ki67+) and nuclei lacking brown stain (blue; Ki67-) were counted. A minimum of 150 nuclei were counted on each image. Replicates for each tissue section or chamber were averaged.

### Statistical analysis

Data were assessed for heterogeneity of variance using Bartlett’s test; data were log-transformed when Bartlett’s test yielded a significance of < 0.05. Data were then assessed by ANOVA. ANOVA with one repeated measure was used for cell culture experiments to reflect repeated use of cells from an individual woman in all treatments in vitro. For ANOVA, post hoc analyses were performed using Duncan’s multiple range test. Statistical analyses were performed using StatPak v4.12 software (Northwest Analytical, Portland, OR). Data are presented as mean + SEM, *n* = 3–5 monkeys or women per treatment group or time point, and significance was assumed at *p* < 0.05.

## Results

### Identification of gonadotropin- and prostaglandin-sensitive gene products

In vivo exposure to an ovulatory gonadotropin stimulus significantly altered granulosa cell expression of 32 individual gene products. Granulosa cell mRNA levels present after ovarian stimulation and 36 h of exposure to hCG (36 h hCG) were compared with granulosa cell mRNA levels present after ovarian stimulation in the absence of hCG (0 h hCG). This comparison yielded 31 mRNAs significantly increased by hCG treatment and 1 mRNA significantly decreased by hCG treatment (Table 2).

**Table 2.** Gonadotropin and celecoxib regulated genes in monkey granulosa cells.

Gene Symbol	Fold Change 36 h hCG/0 h hCG	Fold change 36 h hCG+celecoxib/36 h hCG	Transcript Name
HIST1H2BL	−2.872	+1.547	histone cluster 1, H2bl
APCDD1	+3.260	−1.071	adenomatosis polyposis coli down-regulated 1
PTGES	+3.079	−1.038	prostaglandin E synthase
ADAMTS9	+2.776	−1.045	ADAM metallopeptidase with thrombospondin type 1 motif, 9
FCGR2A	+2.695	+1.396	Fc fragment of IgG, low affinity IIa, receptor (CD32)
DHRS9	+2.591	+1.013	dehydrogenase/reductase (SCD family) member 9
ENPP1	+2.387	+1.001	ectonucleotide pyrophosphatase/phospodiesterase 1
OLFM4	+2.381	−1.007	olfactomedin 4
CRYAB	+2.376	−1.109	crystallin, alpha B
RUNX2	+2.370	−1.062	runt-related transcription factor 2
MOCOS	+2.300	−1.076	molybdenum cofactor sulfurase
PRLR	+2.296	+/−1.000*	prolactin receptor
ANKRD22	+2.254	+1.346	ankyrin repeat domain 22 (transcription regulator)
EREG	+2.185	+1.050	epiregulin (growth factor)
NNMT	+2.143	−1.366	nicotinamide N-methyltransferase
GPC4	+2.135	−1.100	glypican 4
SHC4	+2.119	−1.122	SHC (Src homology 2 domain containing) family, member 4
ABCA9	+2.087	+1.019	ATP-binding cassette, subfamily A (ABC1), member 9
AP1S2	+2.082	−1.009	adaptor-related protein complex 1, sigma 2 subunit
FSTL3	+2.072	−1.055	follicatin-like 3 (secreted glycoprotein)
LGALS3	+2.069	−1.038	lectin, galactoside-binding, soluble, 3
TNFAIP6	+2.067	+1.201	tumor necrosis factor, alpha-induced protein 6
GXYLT2	+2.066	−1.064	glucoside xylosyltransferase 2
FAM129B	+2.065	+1.091	family with sequence similarity 129, member B
HSD11B1	+2.051	+1.046	hydroxysteroid (11-beta) dehydrogenase 1
DIRAS3	+2.048	−1.094	DIRAS family, GTP-binding RAS-like 3
MAP3K13	+2.047	−1.073	mitogen-activated protein kinase kinase kinase 13
VNN1	+2.044	+1.226	vanin 1
SLC16A10	+2.032	−1.046	solute carrier family 16, member 10 (aromatic amino acid transporter)
GFPT2	+2.018	+1.002	glutamine-fructose-6-phosphate transaminase 2
CACNA1E	+2.017	−1.089	calcium channel, voltage-dependent, R type, alpha 1E subunit
COBLL1	+2.001	−1.180	cordon-bleu WH2 repeat protein-like 1

Genes with significantly different expression (±2-fold) for hCG-treated versus untreated granulosa cells (36 h/0 h; left) and the corresponding fold changes for 36 h hCG+celecoxib-treated versus 36 h hGC-treated granulosa cells (36 h+celecoxib/36 h; right). *denotes no change.

The ovulatory gonadotropin surge initiates all ovulatory changes in the primate follicle. One key ovulatory change is the initiation of prostaglandin synthesis, resulting in prostaglandin accumulation 24–36 h after hCG administration, which we have previously reported (Duffy, Dozier, et al. 2005). Note that follicle health is dependent on continued gonadotropin stimulation. It is not possible to determine the impact of PGE2 by simply administering PGE2 in the absence of hCG. Instead, to determine if a subset of all hCG-induced changes are due to elevated follicular prostaglandins, additional monkeys underwent ovarian stimulation followed by 36 h treatment with hCG and the PTGS2 inhibitor celecoxib, which has previously been shown to reduce hCG-stimulated PGE2 (Seachord et al. 2005). Granulosa cell expression of the histone cluster protein HIST1H2BL was significantly reduced by 36 h hCG treatment and somewhat, but not significantly, increased with celecoxib treatment (Table 2). Prostaglandin-sensitive expression of a single gene seemed insufficient to explain the fact that blockade of prostaglandin synthesis results in ovulation failure. So, pathway analysis was performed to identify candidate pathways which are regulated by hCG and prostaglandins at levels other than gene transcription.

### Identification of pathways regulated by gonadotropins and prostaglandins

**Figure 1.**
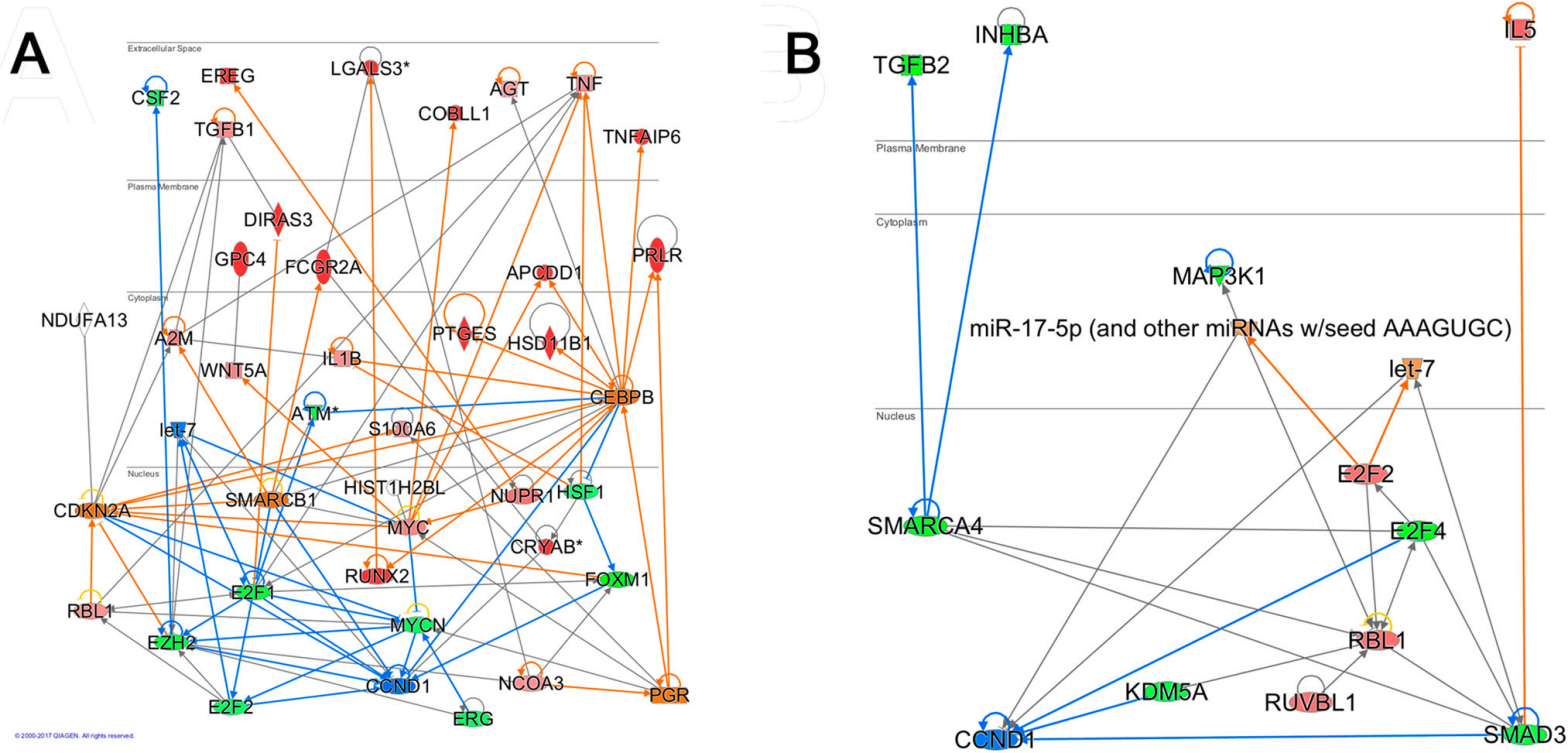
Predicted granulosa cell gene networks regulated by hCG and celecoxib. Top up-regulated genes and down-regulated genes (TG) and IPA-predicted change in upstream regulator (UR) gene activity when comparing 36 h hCG versus 0 h hCG (Panel A) and 36 h hCG + celecoxib versus 36 h hCG (Panel B). Transcripts were included where TG with a change above absolute 2 (Table 1) and predicted UR with an estimated significance below *p*=0.05/Z-score > ABS(2.1) (Table 2). For clarity, p53 and Rb (purple) were not included in the network build as they connected with most nodes in Panel A. Shown is the subcellular view option in IPA, with nuclear localization is at the bottom and extracellular/secreted proteins are at the top; cytoplasmic and plasma membrane proteins are located as indicated. Transcript icons and color scheme are IPA defaults. Briefly, red, orange and yellow are up-regulated, blue and green are down-regulated, and gray are unknown/no prediction as either actual or predicted depending on molecule. Overlays and predictions (provided by the Molecule Activity Predictor function in IPA) were done using transcript expression values for the respective comparison analysis.

IPA was used to generate the networks showing the differential mRNA expression between treatment with 36 h hCG and 0 h hCG (Figure 1(A)). IPA was also used to identify networks showing changes between treatment with 36 h hCG + celecoxib treatment and 36 h hCG only (Figure 1(B)). For each comparison, the total list of differentially-regulated genes (Table 2) as well as IPA predicted upstream regulators (Table 3) were connected to generate the networks. In addition, colors indicating the direction of change in mRNA expression were overlaid for each panel in Figure 1. IPA’s Molecule Activation Predictor function colorizes network connections as blue or orange when gene expression changes are consistent with known gene relationships, and yellow when the relationship is inconsistent. In both panels, numerous blue and orange connections highlight the importance of cell cycle regulation and pro-inflammatory responses within these networks.

**Table 3.** Upstream regulators predicted to be significantly regulated with hCG treatment (36 h hCG/0 h hCG) or with celecoxib treatment (36 h hCG+celecoxib/36 h hCG) based on experimental data.

Molecule Type	36 h hCG/0 h hCG		36 h hCG+celecoxib/36 h hCG	
	Activated	Inhibited	Activated	Inhibited
Growth Factor	TGFB1, AGT		IL5	TGFB2, INHBA
Kinases	ATM			MAP2K1, MAP3K1
Transcription Regulators	CDKN2A, NUPR1, SMARCB1, HSF1, RB1, PPRC1, CEBPB, TP53, EZH2, *RBL1, ERG*	*CCND1*, MYCN, MYC, FOXM1, NCOA3, *E2F2*, E2F1	*E2F2*, *CCND1*,E2F4	SMAD3, SMARCA4, *ERG, RBL1*, KDM5A, RUVBL1
MicroRNA	*let-7*		miR-17-5p	*let-7*
Enzymes	NDUFA13			
Cytokines	TNF, CSF2, *WNT5A*, OSM, IL1A, IL1B	IL21		*WNT5A*
Other	PGR, SHH, A2M	*S100A6*, SFTPA1, PCM1	*S100A6*, EIF4G1	CD24, IGF1R, IGFBP2

*Italics* indicates regulators which differ in both groups, but note that the direction of regulator activity change differs in all cases, meaning that celecoxib counters the directional regulation by hCG.

### Granulosa cell proliferation is controlled by gonadotropin and prostaglandins

Many of the upstream regulators predicted by pathway analysis of our array data (Table 3) are associated with cell cycle regulation. Importantly, three of these predicted upstream regulators (e.g. E2F2, RBL1, CCND1) are implicated in control of cell cycle progression (Johnson and Walker 1999). Therefore, cell cycle regulation was selected for further analysis.

**Figure 2.**
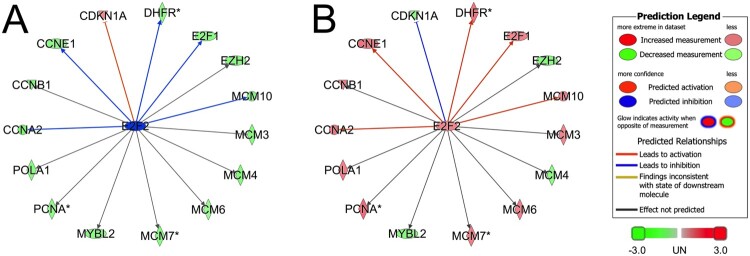
E2F2 is a predicted node for regulation of granulosa cell cycle modulators. Panel A. Comparison of 36 h hCG-treated vs untreated granulosa cells (36 h / 0 h) showed IPA predicted down-regulation (green) as the primary effect of the predicted inhibition (blue) of E2F2. Panel B. Comparison of 36 h hCG + celecoxib versus 36 h hCG in vivo (36 h + celecoxib/36 h) showed IPA predicted predominantly up-regulation (red) as the effect of the predicted activation (orange) of E2F2 in granulosa cells.

A network of E2F2-regulated genes was overlaid with ratios of mRNA expression data from 36 h hCG/0 h hCG and 36 h hCG + celecoxib/36 h hCG (Figure 2). These comparisons were selected because they are physiologically meaningful. The ratio of 36 h hCG/0 h hCG reflects changes stimulated by hCG as the follicle transitions from a dominant preovulatory follicle to a luteinizing follicle just before ovulation (Figure 2(A)). Treatment with hCG decreases expression of many cell cycle regulators, as denoted by the green color. Importantly, the majority of these proteins increase the rate of cell proliferation. In addition, hCG increased expression of CDKN1A, an inhibitor of cell cycle progression. It is important to note that the overall impact of hCG is to subtly, but not significantly, alter the mRNA expression of many cell cycle regulators, with the net effect of decreasing cell proliferation.

In contrast, the ratio of 36 h hCG + celecoxib/36 h hCG shows the impact of prostaglandin depletion in the ovulatory follicle (Figure 2(B)). The overlay shows that prostaglandin depletion effectively reverses the effect of hCG, upwardly shifting expression of many gene products which increase cell proliferation and downwardly shifting expression expression of a cell cycle inhibitor, CDKN1A. These data demonstrate that the role of hCG-stimulated prostaglandins is to subtly shift cell cycle to decrease the rate of cell proliferation. However, no single mRNA is significantly increased or decreased by prostaglandins.

E2F2 is the predicted locus of control for this pathway (Figure 2). IPA predicted that an ovulatory dose of hCG decreases E2F2 activity as a control point for shifting granulosa cells from a highly proliferative state to a state of very limited cell cycle progression. Furthermore, this analysis predicted that prostaglandins (stimulated by hCG) subtly alter mRNA expression of this same cluster of gene products to decrease cell cycle progression by via the activity of E2F2.

**Figure 3.**
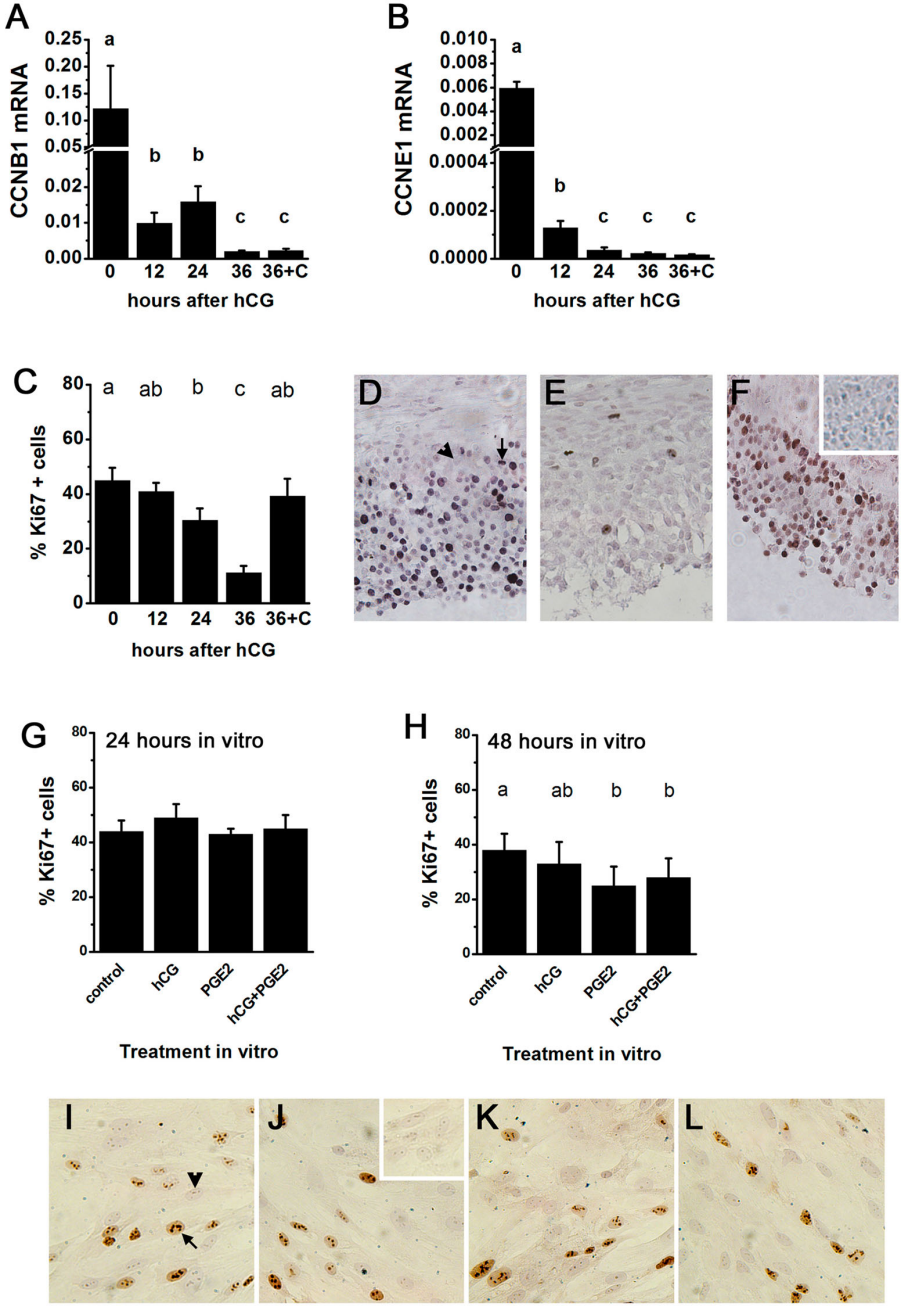
hCG and PGE2 regulate granulosa cell proliferation. Panels A-B. *CCNB1* (A) and *CCNE1* (B) levels in monkey granulosa cells obtained after ovarian stimulation without (0 h) hCG or 12, 24, or 36 h after administration of hCG; additional monkeys received hCG and celecoxib for 36 h (36+C). mRNA of interest was expressed relative to *ACTB*. Panel C. The percentage of Ki67 immunopositive granulosa cells in monkey ovarian tissues obtained after treatments described for Panels A-B. For each panel A-C, groups with no common superscripts are different by ANOVA and Duncans post hoc test, *p*<0.05; n=3–5 monkeys/time or treatment. Panels D-F. Representative images of Ki67 immunodetection in monkey granulosa cells (lower portion of each image) obtained after treatment with 0 h hCG (D), 36 h hCG (E), and 36 h hCG + celecoxib (F). Example Ki67+ (arrow) and Ki67- (arrowhead) nuclei are indicated in Panel D; inset in panel F shows lack of stain in granulosa cells when primary antibody was omitted. Panels G-H. Human granulosa cells received indomethacin alone (control) or in combination with hCG, PGE2, or hCG + PGE2 for 24 h (G) or 48 h (H) before Ki67 immunodetection; *n* = 4 women/treatment. For each panel G-H, groups with no common superscripts are different by ANOVA with one repeated measure (for repeated use of each woman’s cells) and Duncans post hoc test, *p* < 0.05. Panels I-L. Representative images of Ki67 immunodetection in human granulosa cells cultured for 48 h with control media (I), hCG (J), PGE2 (K), or hCG + PGE2 (L). Example Ki67+ (arrow) and Ki67- (arrowhead) nuclei are indicated in panel I. Inset in panel J shows lack of stain in granulosa cells when primary antibody was omitted.

Experiments confirmed that mRNA levels for two cell cycle regulators, CCNB1 and CCNE1, decreased after exposure to hCG in vivo (Figure 3(A,B)). Treatment with 36 h hCG + celecoxib did not alter *CCNB1* and *CCNE1* levels when compared to treatment with 36 h hCG only (Figure 3(A,B)). These findings are consistent with gene expression levels in Figure 2. In this Figure, inhibition of cell cycle regulation was seen in the 36 h hCG/0 h hCG ratio and activation of cell cycle regulation was seen in the 36 h hCG + celecoxib/36 h hCG ratio, albeit subtle.

Additional prospective studies were conducted to test the hypothesis that prostaglandins mediate hCG-regulated reduction in cell cycle progression by follicular granulosa cells. Ki67 immunodetection was performed to determine the percentage of monkey granulosa cells engaged in cell cycle progression before hCG treatment (0 h hCG), at specific times after hCG administration, and after treatment with 36 h hCG + celecoxib in vivo (Figure 3(C–F)). Before hCG administration, 45 ± 5% of granulosa cells were Ki67 + . The percentage of Ki67+ granulosa cells was significantly reduced 24 h after hCG administration (31 ± 4%), and only 11 ± 3% of granulosa cells were Ki67+ by 36 h after hCG administration. Co-administration of hCG and celecoxib yielded 39 ± 6% of granulosa cells determined to be Ki67+, similar to the percentage of Ki67+ granulosa cells observed at 0 h hCG. To determine if the specific prostaglandin PGE2 is involved in cessation of cell cycle progression, human granulosa cells were maintained for 7 days in vitro using culture conditions which restore responsiveness to gonadotropin stimulation. Granulosa cells were treated in vitro with the general cyclooxygenase inhibitor indomethacin to block endogenous prostaglandin production; PGE2 was replaced in some cultures in the absence or presence of hCG. Culture for 24 h did not reveal differences in Ki67 immunodetection between treatments (Figure 3(G)). However, cultures treated in vitro for 48 h in vitro showed that PGE2, but not hCG, reduced the percentage of Ki67 immunopositive cells (Figure 3(H–L)).

## Discussion

The overall goal of the present study was to utilize array technology and pathway analysis to identify gene products and gene networks utilized by PGE2 to mediate ovulation and luteinization. Previous studies showed that PGE2 levels in primate follicles are elevated during the second half of the interval between the ovulatory gonadotropin surge and ovulation (Duffy and Stouffer 2001). The present analysis included identification of gene products which were different before and 36 h after hCG administration, just before the expected time of ovulation. Co-administration of hCG and the PTGS2 inhibitor celecoxib was previously shown to prevent rising intrafollicular prostaglandins in monkeys (Seachord et al. 2005). If hCG-stimulated prostaglandins are important regulators of a gene product within the follicle, then it was anticipated that hCG would increase and hCG + celecoxib would decrease expression levels. The converse (expression decreased by hCG and expression increased by hCG + celecoxib) would also indicate a role for prostaglandins to mediate hCG-regulated gene expression.

*HIST1H2BL* was significantly decreased with hCG, but increased *HIST1H2BL* with hCG + celecoxib did not meet the significance criteria established for this study. A previous gene array study identified HIST1H2BL as a hCG-regulated gene in granulosa cells of human ovulatory follicles (Wissing et al. 2014). As a core histone protein, HIST1H2BL facilitates DNA replication and cell proliferation (Marzluff and Duronio 2002). Decreased *HIST1H2BL* expression after hCG exposure would contribute to decreased cell cycle progression. Subtle, elevated expression with hCG + celecoxib is consistent with a role for prostaglandins as key local mediators of a cluster of genes which, acting together, contribute to granulosa cell exit from cell cycle. However, a subtle change in the expression of a single gene is unlikely to be responsible for the major, prostaglandin-dependent changes observed during ovulation in vivo.

Network analysis predicted that cell cycle regulation is a major hCG- and prostaglandin-controlled process in granulosa cells of ovulatory follicles. Celecoxib treatment was predicted to alter expression and/or activity of the cell cycle regulators E2F2, RBL1, and CCND1 in a manner consistent with the hypothesis that hCG decreases cell cycle progression in a prostaglandin-dependent manner. The RBL1 protein is thought to bind to E2F2 and other members of the E2F family of transcription regulators to decrease transcription of cell cycle regulators and other gene products involved in cell cycle progression (Johnson and Walker 1999). This includes regulation of CCND1 (also known as cyclin D1), which regulates the transition from the G1 to S phase of the cell cycle (Johnson and Walker 1999). The concept that the transcription regulator E2F2 serves as a control point for cell cycle regulation specifically in granulosa cells is supported by the linkage of E2F2 to expression of many cell cycle regulators, also shown to be modestly up- or down-regulated in monkey granulosa cells. This finding is complementary to a recent report of network analysis in human granulosa cells without and with hCG, where all networks with predicted decreases in activity with hCG were related to cell cycle (Wissing et al. 2014). Indeed, this analysis of human granulosa cells included many of the specific gene products identified in the present study. It is interesting to note that many genes predicted to be regulated by E2F2 were altered in a direction consistent with hCG + prostaglandin-reduced cell cycle activity, even though the modest change in the expression level of each of these genes did not meet the stringent significance criteria for changes in mRNA levels set for this study. However, these finding are consistent with the hypothesis that prostaglandin stimulation leads to small changes in levels of mRNA, protein, and/or activity of many cell cycle regulators, and combined contributions of these many small changes cause an overall decreased the rate of cell cycle progression.

The prediction that a prostaglandin, and in particular PGE2, is involved in granulosa cell withdrawal from cell cycle was tested directly in vivo and in vitro. Administration of hCG in vivo caused a decrease in the percentage of granulosa cells in active cell cycle, as determined by immunodetection of Ki67. These data are consistent with previous reports showing that granulosa cells exit cell cycle in the hours following the ovulatory gonadotropin surge (Robker and Richards 1998; Chaffin et al. 2001). In contrast, co-administration of hCG and celecoxib for 36 h failed to cause granulosa cell exit from cell cycle. This finding indicates that a PTGS2 product is necessary for hCG to reduce proliferation in granulosa cells. Our in vitro studies of human granulosa cells identified PGE2 as the key prostaglandin involved in cell cycle regulation. Previous reports find limited evidence of a correlation between prostaglandins and granulosa cell proliferation (Wang et al. 1992; Li J et al. 1996; Mori et al. 2011; Li F et al. 2012), but there was no consensus as to whether prostaglandins promote or inhibit cell cycle progression. Some studies demonstrate that granulosa cell exit from cell cycle begins before prostaglandins accumulate in the follicle (Mikuni et al. 1998; Duffy and Stouffer 2001), indicating that the ovulatory gonadotropin surge is likely the initial stimulus for granulosa cell cycle exit. However, the present study shows that a PTGS2 product is needed for cell cycle exit in vivo, and PGE2 is able to stimulate primate granulosa cell exit from cell cycle in vitro. While gonadotropin likely initiates cell cycle exit in vivo, gonadotropin-stimulated production of PGE2 is needed to achieve and maintain very low rates of granulosa cell proliferation which occur at the time of ovulation and luteal formation.

Cessation of proliferation is a prerequisite for differentiation from the follicular granulosa cell phenotype to the luteal cell phenotype (reviewed in (Robker and Richards 1998)). Functional luteinization, as defined by elevated progesterone production, is initiated by gonadotropin, independent of prostaglandins, in non-human primates and women (Duffy 2015). Structural luteinization includes granulosa cell hypertrophy to become large luteal cells, formation of a capillary network within the granulosa cells, and replacement of the antral space with a mature corpus luteum; these changes do not occur when prostaglandin synthesis is inhibited during exposure to an ovulatory dose of gonadotropin (Kim et al. 2014; Trau et al. 2015). A critical role for follicular prostaglandins in granulosa cell cycle exit is consistent with the formation of luteinized unruptured follicles. In women with endogenous LH surges or receiving hCG to initiate ovulatory events, co-administration of a prostaglandin synthesis inhibitor caused the formation of luteinized unruptured follicles (Killick and Elstein 1987; Pall et al. 2001; Jesam et al. 2010). These follicles continue to increase in diameter after the LH surge or hCG and can remain as fluid-filled, cystic structures for a week or more after an ovulatory dose of gonadotropin. Similar studies in non-human primates and cows used intrafollicular administration of a prostaglandin synthesis inhibitor to prevent elevated follicular prostaglandins after administration of a ovulatory dose of hCG. These treatments resulted in formation of enlarged, cystic structures with multiple layers of unluteinized, proliferative granulosa cells (Peters et al. 2004; Kim et al. 2014). Normal or near-normal serum progesterone levels measured in these studies support the concept that functional luteinization occurs in the absence of prostaglandins. Elevated intrafollicular prostaglandins are involved in structural luteinization and contribute to this process, at least in part, by stimulating granulosa cell exit from cell cycle.

The study presented here used non-human primate granulosa cell RNA obtained after specific conditions of hCG administration and prostaglandin depletion to facilitate identification of a previously-unappreciated network of prostaglandin-sensitive granulosa cell gene products. However, this approach did not identify many granulosa cell mRNAs previously shown to be hCG-regulated in human and non-human primate ovarian cells (Xu et al. 2011; Wissing et al. 2014; Yerushalmi et al. 2014). Oocytes and surrounding cumulus were manually removed from our follicular aspirates, so the cells used for gene array analysis in the present study were thought to be primarily (but not exclusively) mural granulosa cells. Prostaglandin-regulated gene products implicated in cumulus expansion in rodents (reviewed in (Russell and Robker 2007)) were likely not identified in the present study since many cumulus cells were removed prior to preparation of cDNA for analysis. Expression levels for many mural granulosa cell gene products increase immediately after the LH surge or hCG administration and are low again by the time of ovulation in primates (Chaffin et al. 1999, 2000; Duffy, Dozier, et al. 2005). mRNAs regulated in this fashion would not be detected by our analysis. Focused analysis of changing patterns of gene expression late in the ovulatory interval allowed identification of cell cycle regulation as a PGE2-sensitive process that is essential for both ovulation and formation of a fully-functional corpus luteum.

## Availability of data and materials

The datasets generated and/or analyzed during the current study are not publicly available due intellectual property considerations but are available from the corresponding author on reasonable request.
